# Mechanisms of Beta-Cell Apoptosis in Type 2 Diabetes-Prone Situations and Potential Protection by GLP-1-Based Therapies

**DOI:** 10.3390/ijms22105303

**Published:** 2021-05-18

**Authors:** Safia Costes, Gyslaine Bertrand, Magalie A. Ravier

**Affiliations:** IGF, Univ. Montpellier, CNRS, INSERM, 34094 Montpellier, France; gyslaine.bertrand@igf.cnrs.fr

**Keywords:** pancreatic beta-cells, islets, apoptosis, islet amyloid, lipotoxicity, glucotoxicity, GLP-1

## Abstract

Type 2 diabetes (T2D) is characterized by chronic hyperglycemia secondary to the decline of functional beta-cells and is usually accompanied by a reduced sensitivity to insulin. Whereas altered beta-cell function plays a key role in T2D onset, a decreased beta-cell mass was also reported to contribute to the pathophysiology of this metabolic disease. The decreased beta-cell mass in T2D is, at least in part, attributed to beta-cell apoptosis that is triggered by diabetogenic situations such as amyloid deposits, lipotoxicity and glucotoxicity. In this review, we discussed the molecular mechanisms involved in pancreatic beta-cell apoptosis under such diabetes-prone situations. Finally, we considered the molecular signaling pathways recruited by glucagon-like peptide-1-based therapies to potentially protect beta-cells from death under diabetogenic situations.

## 1. Introduction

Type 2 diabetes (T2D) is characterized by chronic hyperglycemia due to an insufficient insulin secretion to effectively lower plasma glucose concentrations in the context of insulin resistance of target tissues. The amount of released insulin depends on the output of each beta-cell from pancreatic islets of Langerhans (beta-cell function) and of the total number of these cells (beta-cell mass). There is evidence that beta-cells have a first compensatory phase to counteract insulin resistance by increasing insulin secretion to maintain euglycemia [1,2]. Indeed, hypersecretion of insulin has been reported in obese patients [3,4,5], and the beta-cell mass was shown to be increased in obese non-diabetic individuals [6,7,8] and in insulin resistant patients [9] through neogenesis rather than proliferation [6,9,10], and/or transdifferentiation of both acinar/ductal cells and alpha-cells into beta-cells (see reviews [2,11]). Since insulin secretion measured in vivo [3,4,5] cannot be correlated to beta-cell mass in the same patient [6,7,8,9], it is difficult to investigate their respective contribution in that context, but both seem to contribute to the insulin compensatory increase. Whereas beta-cells seem to compensate for high insulin demand that occurs in obesity, when the compensation mechanisms are lost and beta-cells become exhausted, hyperglycemia appears [1,2].

The alteration in glucose-induced insulin secretion in human T2D was reported to result from beta-cell dysfunction associated or not with a decrease in beta-cell mass. A loss of first- and reduced second-phase insulin responses [12] with alteration of insulin oscillatory release [13,14] are well established beta-cell functional abnormalities in T2D. Beta-cell function in patients with T2D was reported to be reduced by 50% at diagnosis [15] while the beta-cell mass was only reduced by 24% [7]. Since patients undergoing hemi pancreatectomy for donation to a relative with type 1 diabetes showed normal 24-h glucose profiles [16], and deterioration of insulin secretion and glucose tolerance one year later [16] with an increased risk of developing T2D only in the presence of obesity and insulin resistance [17], the contribution of the decreased beta-cell mass to the onset of T2D appeared as a subject of debate [6,7].

Beta-cell mass cannot yet be accurately measured in living patients; therefore, our knowledge relies on pancreatic tissue sections from autopsies. A significant reduction in beta-cell mass by 40–65% in subjects with glucose intolerance [6] and in T2D patients [6,7] compared to nondiabetic subjects matched for body mass index (BMI) has been observed, and was also reported by other studies including fewer subjects [18,19,20]. If the involvement of the decrease in beta-cell mass in T2D onset is still controversial, its gradual decline with duration of the disease undoubtedly contributes to the progressive deterioration of glucose homeostasis [7]. Of note, it was also recently commented that persons with and without T2D can have a similar beta-cell mass, but because of huge variabilities in insulin sensitivity and insulin secretion in the general population, the total mass is inadequate and might be responsible for their diabetes [21].

Several possibilities have been highlighted to explain the default in beta-cell mass in T2D, such as a low innate beta-cell mass [22], a failed increase in beta-cell mass in response to insulin resistance [2,6] or senescence [23], and/or a progressive beta-cell loss caused by apoptosis [6] or beta-cell dedifferentiation [24]. Recent studies also suggested the involvement of beta-cell ferroptosis, a nonapoptotic regulated cell death that relies on iron-dependent regulated necrosis [25].

**Figure 1:**
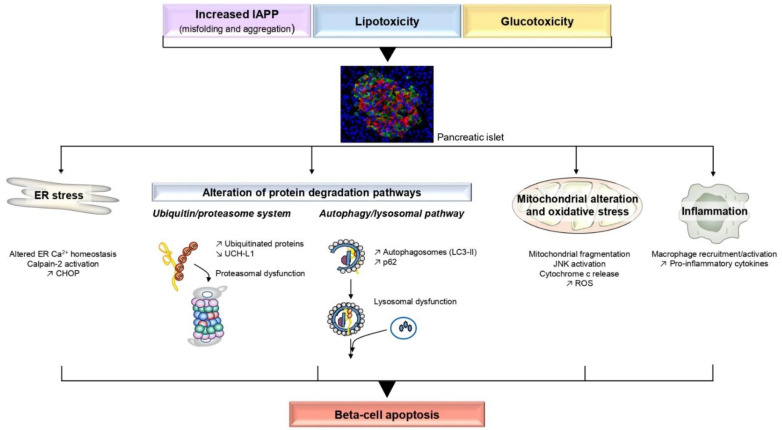
Main mechanisms involved in pancreatic beta-cell apoptosis under T2D-prone situations. Increased islet amyloid polypeptide (IAPP) levels with misfolding and aggregation, lipotoxicity and glucotoxicity are the most investigated causative factors of beta-cell demise. These situations individually elicit stress pathways such as endoplasmic reticulum (ER) stress, mitochondrial/oxidative stress, inflammation, and disrupt the main pathways of protein clearance (ubiquitin-proteasome system and autophagy/lysosomal pathway). The synergistic deleterious effects of these situations as well as the crosstalk between the stress pathways ultimately contribute to beta-cell apoptosis. The immunofluorescence image is a human islet showing beta-cells in red and alpha-cells in green.

**Figure 2:**
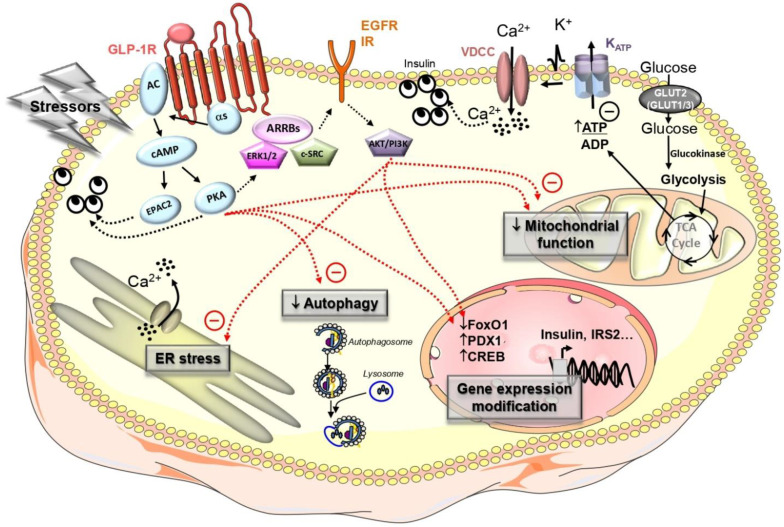
Main molecular mechanisms induced by glucagon-like peptide-1 (GLP-1) to protect beta-cells from apoptosis. Beta-cell stressors (such as increased IAPP, gluco- and/or lipotoxicity) may trigger ER stress, alter mitochondrial function and/or the autophagic flux and modify gene expression. GLP-1 and GLP-1RA have been reported to protect beta-cells by alleviating these deleterious effects.

The purpose of this review is to discuss recent insights into the molecular mechanisms involved in beta-cell apoptosis in T2D. Indeed, several studies have described a significant increase of beta-cell apoptosis in sections of pancreas as one plausible cause for the decreased beta-cell mass in T2D [6,10,26,27]. Supporting this concept, beta-cell apoptosis was also evidenced in isolated human T2D islets [27,28,29]. As stated above, given the difficulty to assess beta-cell mass in vivo in humans [1], most studies have been performed post mortem or in animal models of T2D, although mouse and human beta-cells may behave differently. Additionally, it should also be stressed that the recourse to isolated pancreatic islets and cultured beta-cells (clonal and primary) is still required to elucidate the molecular mechanisms involved in beta-cell apoptosis due to limited availability of human samples and shortage of technologies. This review will focus on the molecular mechanisms reported to alter beta-cell survival under T2D-prone situations, such as amyloid deposits, lipotoxicity and glucotoxicity. Each situation will be reviewed in separate sections, but it should be borne in mind that in the pathophysiological context they undoubtedly exert synergistic effects. Moreover, if each individual alteration such as endoplasmic reticulum (ER) overload, oxidative stress, inflammation, etc., may not lead to immediate apoptosis in vivo, their cumulative effects will exacerbate the deleterious outcome of each pathway over time (Figure 1). We will also review whether glucagon-like peptide-1 (GLP-1) based therapies can influence beta-cell apoptosis in the context of T2D (Figure 2).

## 2. Molecular Mechanisms Involved in Beta-Cell Apoptosis

### 2.1. Islet Amyloid Polypeptide

The islet in T2D is characterized by amyloid deposits derived from islet amyloid polypeptide (IAPP), a protein synthesized and secreted along with insulin by pancreatic beta-cells. Due to an amyloidogenic sequence, IAPP has the propensity to form oligomers and subsequently insoluble fibrils in species at risk to develop diabetes (cats, primates and humans) [22]. Indeed, islet amyloid has frequently been reported in islets from patients with T2D [18,20,30]. Further supporting a role of IAPP in the development of human T2D, a rare missense mutation in the IAPP gene (S20G) that increases its amyloidogenicity [31], is associated with beta-cell deficit and increased risk for T2D [32].

In rodents, IAPP is nonamyloidogenic due to proline substitutions in the amyloidogenic sequence, and these species do not spontaneously develop diabetes [33]. However, overexpression of human IAPP (h-IAPP) in rodent models promotes amyloid deposits, beta-cell dysfunction and apoptosis, consequently leading to reduced beta-cell mass and hyperglycemia [22,33,34], supporting the role of h-IAPP as a contributor to the islet pathology in human T2D. The following paragraphs will focus on the molecular pathways by which amyloidogenic IAPP promotes beta-cell apoptosis.

#### 2.1.1. ER Stress and Aberrant Ca^2+^ Release

ER stress is a well-established feature of T2D [35,36,37] that is induced by accumulation of misfolded/unfolded proteins in the lumen of the ER. To prevent the deleterious consequences of ER stress, an unfolded protein response (UPR) is engaged by the cells with activation of three main branches of signaling transducers: inositol requiring ER-to-nucleus signal kinase 1 (IRE1), PKR-like ER kinase (PERK) and activating transcription factor 6 (ATF6), leading to attenuation of global protein translation, synthesis of folding enzymes and ER-associated degradation. Even if ER overload does not necessarily culminate in apoptosis, severe prolonged and unresolved ER stress can shift the balance towards proapoptotic pathway activation.

To face insulin resistance, healthy beta-cells will secrete larger amount of insulin. Since IAPP is coexpressed with insulin, this compensatory production/hypersecretion of insulin and IAPP may exceed the synthesis, folding and trafficking capacity of the ER, eventually leading to the formation of membrane-permeant toxic oligomers [38]. Indeed, toxic oligomers of IAPP have been found associated with ER membranes in beta-cells of individuals with T2D [38], likely contributing to ER stress. Despite one study describing that amyloid formation was not associated with significant increase in ER stress markers [39], most studies performed in transgenic rodent overexpressing h-IAPP, rodent beta-cell lines and human islets/pancreases agree that oligomerization-prone h-IAPP induces ER stress-mediated beta-cell apoptosis [35,40,41,42]. In support of this postulate, deletion of the ER stress marker C/EBP homologous protein (CHOP) was shown to delay beta-cell loss and diabetes onset in h-IAPP transgenic mice [43]. However, deletion of CHOP only partially prevents mice from h-IAPP-induced diabetes [43], suggesting that other molecular mechanisms are involved in h-IAPP toxicity.

Given their membrane-disrupting properties, h-IAPP oligomers may trigger physical changes in the plasma membrane that can be sensed by nonselective ion channels leading to aberrant cytosolic free Ca^2+^ concentration increases [44,45]. Additionally, since toxic oligomers were found to be associated with intracellular membranes of ER, secretory vesicles and mitochondria in beta-cells of T2D subjects [38], we cannot exclude the possibility that local membrane instability caused by toxic oligomers permits unregulated Ca^2+^ release from the ER. Indeed, overexpression of h-IAPP, leading to the formation of toxic oligomers, induced apoptosis through increased cytosolic Ca^2+^ and activation of the Ca^2+^-dependent proapoptotic protease calpain-2 in INS-1 832/13 beta-cells and isolated human islets [45]. The detection of cleaved alpha-spectrin, a target of calpain-2 and indicator of a compromised cytoskeleton and cellular membranes, in beta-cells from T2D subjects further indicates that calpain may play a key role in the pathophysiology of T2D [45]. In line with this assumption, suppression of calpain activation attenuates h-IAPP-induced beta-cell apoptosis in human islets [45] and in a transgenic mouse model, thereby preventing diabetes onset [46].

#### 2.1.2. Alteration of Protein Degradation Pathways

To prevent ER stress-induced apoptosis, cells also promote the elimination of misfolded proteins by the ER-associated degradation. In the cytoplasm, protein quality control is achieved by the ubiquitin-proteasome pathway that involves recognition of dysfunctional/misfolded proteins, their covalent conjugation to ubiquitin and subsequent degradation by the proteasome. Increased expression of amyloidogenic h-IAPP in beta-cells alters the ubiquitin–proteasome system as shown by the accumulation of polyubiquitinated proteins in vitro in clonal beta-cells and isolated human islets, but also in vivo in h-IAPP transgenic rodents [40,41,47]. We further demonstrated that this accumulation of ubiquitinated proteins is due to a deficit in ubiquitin C-terminal hydrolase L1 (UCH-L1), a deubiquitinating enzyme that allows ubiquitinated proteins to access the proteasome [47]. Importantly, deficit in UCH-L1 enhances ER stress-induced apoptosis in INS-1 832/13 cells and in vivo in h-IAPP transgenic mouse beta-cells [47,48]. The potential involvement of this deleterious mechanism in T2D was evidenced by the presence of polyubiquitinated proteins and decreased UCH-L1 levels in beta-cells of subjects with T2D [47].

The ubiquitin–proteasome system is not the unique pathway involved in the elimination of misfolded proteins. The autophagy/lysosomal pathway (or macroautophagy) also plays a key role to prevent the intracellular accumulation of misfolded/aggregated proteins and damaged organelles. This pathway involves the formation of a double-membrane vesicle, the autophagosome, to surround the material to be degraded. The autophagosomes then fuse with lysosomes in which the sequestered material is degraded by hydrolytic enzymes. Whereas one would have expected that the lysosomal degradation will compensate for the compromised ubiquitin–proteasome system under h-IAPP overexpression, the autophagy pathway is rather impaired in beta-cells overexpressing h-IAPP as demonstrated in vivo in h-IAPP transgenic mice and rats [49]. Consequently, the alteration in lysosomal degradation impairs the clearance of damaged mitochondria through mitophagy [50], inducing oxidative stress and further exacerbating beta-cell ER stress and apoptosis. In addition, the implication of the autophagy in h-IAPP clearance itself shown in isolated human islets and in transgenic mouse models [51,52,53,54] contributes to a vicious cycle whereby IAPP reduces lysosomal degradation, which further promotes IAPP overload and toxicity. Further providing evidence of such deleterious mechanism in vivo, a recent article reveals that an autophagy enhancer ameliorated diabetes of h-IAPP transgenic mice through clearance of amyloidogenic oligomers [52]. The potential involvement of autophagy deficits in the decline of beta-cell mass in human T2D has been suggested by the accumulation of autophagic vacuoles [55] and the increased levels of p62, a marker for lysosomal degradation defects, in beta-cells of T2D human islets [37,53,56]. It is of note that dead beta-cells with signs of altered autophagy and no major chromatin condensation observed in T2D patients rather reflect an autophagy-associated cell death [55]. This form of programmed cell death morphologically distinct from apoptosis may therefore additionally contribute to beta-cell loss in T2D.

#### 2.1.3. Oxidative Stress

Oxidative stress, defined as excessive production and accumulation of reactive oxygen species (ROS), is another mediator of h-IAPP-induced beta-cell apoptosis. In autopsy pancreatic tissues from Japanese patients with T2D, beta-cell loss and islet amyloid are associated with expression of oxidative stress markers [19,37]. In vitro and ex vivo experimental research using rodent beta-cell lines, islets isolated from h-IAPP transgenic mice or human islets showed that h-IAPP/islet amyloid induces oxidative stress, thus contributing to beta-cell apoptosis [50,57,58,59]. Involvement of oxidative stress in h-IAPP toxicity was further supported by the reduction in h-IAPP-induced beta-cell death following exposure of h-IAPP mouse islets and rat insulinoma RIN-m5F cells to antioxidant or thiol/disulfide reducing agents [59,60]. Mechanistically, overexpression or exogenous addition of h-IAPP activates apoptosis signal-regulating kinase 1 (ASK1), in rodent clonal beta-cells, in beta-cells from h-IAPP transgenic mice and in human islets, leading to c-Jun N-terminal kinase (JNK) activation and beta-cell apoptosis [57,58]. Inhibition of ASK1 was shown to decrease h-IAPP-induced toxicity in RIN-m5F cells and isolated human islets [57]. Interestingly, h-IAPP-induced JNK activation is a critical downstream mediator in both mitochondria-dependent (intrinsic) and death receptor-mediated (extrinsic) beta-cell apoptosis as reported in the h-IAPP transgenic mouse model [58]. Whereas, the involvement of the intrinsic pathway in the cytotoxicity of h-IAPP is consistent with its role in induction of oxidative stress, the activation of the extrinsic pathway suggests that h-IAPP also plays a role in inflammation.

#### 2.1.4. Inflammation

Amyloidogenic IAPP toxicity is also linked to islet inflammation and macrophage infiltration, characteristics of islet pathology in T2D [61,62]. Indeed, h-IAPP increases the expression of genes encoding chemokines, macrophage markers, nucleotide-binding domain leucin-rich repeat and pyrin-containing receptor 3 (NLPR3) inflammasome components and proinflammatory cytokines in islets from h-IAPP transgenic mice fed with high fat diet [63]. In line with these results, h-IAPP aggregation triggers activation of the NLRP3 inflammasome, leading to the production of the proinflammatory cytokine interleukin 1β (IL1β) from macrophages and dendritic cells in vitro [64] as well as from resident islet macrophages in h-IAPP transgenic mice [65,66]. Whereas the source of islet IL1β remains under consideration (resident macrophages and/or beta-cells themselves), this proinflammatory cytokine is known to induce beta-cell apoptosis. In human islets and islets from h-IAPP transgenic mice, IL1β mediates amyloid-induced apoptosis through upregulation of the cell death receptor Fas and caspase-8 activation [67,68,69]. In addition, amyloid formation reduces the levels of the natural IL1 receptor antagonist (IL1-Ra) in human islets [70], potentially promoting IL1β-induced beta-cell death. Consistent with the involvement of receptor-mediated processes in h-IAPP-induced inflammation, the receptor for advanced glycation end products (RAGE) was also reported to interact with toxic h-IAPP intermediates to mediate inflammation and cytoxicity in INS-1 beta-cells and murine primary islets [71]. Prevention of this interaction in vivo inhibited h-IAPP toxicity and ameliorated islet pathology in h-IAPP transgenic mice [71].

### 2.2. Lipotoxicity

Beta-cells can be exposed to high circulating levels of free-fatty acids (FFAs) coming from dietary origin or released by adipose tissue in the context of obesity, a T2D risk factor. Therefore, in vitro studies have suggested that prolonged exposure (>12 h) to saturated FFAs alters beta-cell function and survival, a phenomenon that was called lipotoxicity. Nevertheless, whether the increase in FFAs levels in vivo is high enough to damage beta-cells is still hotly debated with strong arguments from both sides [72,73,74]. Additionally, it remains unclear to what amount and type of lipids beta-cells are indeed exposed in obese and/or T2D patients. This discussion is beyond the scope of our review, so we decided to summarize the available literature on the in vitro mechanisms underlying the toxic effects of FFAs on beta-cells/islets, in order to highlight potential targets involved in apoptosis that might be relevant in vivo. Palmitate is not the only saturated FFA that may target beta-cells in vivo, but it is the most abundant in human plasma, and it was shown to be positively associated with T2D incidence [75]. In addition, palmitate is more toxic than monounsaturated oleate and polyunsaturated linoleate in clonal rodent beta-cells and dispersed human islet cells [76,77]. In the following paragraphs, we will therefore focus on the molecular mechanisms involved in beta-cell death under chronic palmitate exposure.

#### 2.2.1. ER Stress and Aberrant Ca^2+^ Release

As demonstrated by the upregulation of a large number of UPR genes in human islets [78] and the activation of PERK and IRE1 branches in INS-1E cells, rat primary beta-cells and human islets [79], an ER stress response is induced by chronic exposure to palmitate. IRE1-induced JNK activation and PERK-induced CHOP contribute to the execution of apoptosis in INS-1E cells [79]. Caspase-12, a prodeath protease located on the outer surface of the ER, is subsequently activated to initiate the proapoptotic cascade caspase under lipotoxic stress [79]. To trigger this deleterious ER response, chronic palmitate depletes ER Ca^2+^ stores, therefore leading to PERK activation, alteration in ER Ca^2+^ homeostasis and subsequent ER stress-induced apoptosis in rodent clonal beta-cells and human islets [79,80,81,82]. Another consequence of ER Ca^2+^ depletion is the increase in resting cytoplasmic free Ca^2+^ levels [80], known to initiate a Ca^2+^-dependent beta-cell death pathway along with the activation of calpain-2 as observed in INS-1 832/13 cells treated with palmitate and high glucose, but also in islets from diabetic *db*/*db* mice [82].

Chronic palmitate not only disrupts protein folding capacity of the ER, but also induces ER protein overload. Palmitate alters ER lipid rafts distribution [83] and induces aberrant protein palmitoylation [84], therefore reducing ER-to-Golgi protein trafficking and contributing to beta-cell lipoapotosis [85]. In line with these data, a recent combined human islet transcriptomic and INS-1E cell proteomic study revealed that palmitate modifies genes involved in ER function, ER-to-Golgi transport and ER stress pathway in beta-cells [86]. Initiation of ER stress by palmitate also activates the intrinsic mitochondrial pathway of apoptosis in clonal and primary rat beta-cells, pointing to an ER stress-mitochondrial cross talk involved in lipotoxic beta-cell apoptosis [87].

#### 2.2.2. Mitochondrial Alterations

Palmitate-induced beta-cell apoptosis was shown to be mediated by the intrinsic mitochondrial pathway as demonstrated by the translocation of the proapoptotic component Bax from the cytosol to the mitochondria, and the subsequent cytochrome c release from the mitochondria to form the apoptosome involved in caspase-9 and -3 activation in INS-1E cells [87]. Contributing to the induction of this mitochondrial pathway of apoptosis, in vitro exposure to palmitate also reduces the expression of the antiapoptotic components Bcl-xl and Bcl-2 [87,88], and induces the expression of proapoptotic members death protein 5 (DP5) [87] and p53-upregulated modulator of apoptosis (PUMA) in clonal, primary rat and human beta-cells [87,88]. Supporting their role in palmitate-induced beta-cell apoptosis, knockdown of either DP5 or PUMA reduces apoptosis in rat and human beta-cells and protects mice from high fat diet-induced diabetes [87]. Palmitate further contributes to beta-cell apoptosis through disruption of the mitochondrial network as illustrated by the punctuated/fragmented mitochondria morphology in rodent clonal beta-cells and human islets [78,87,89,90]. In addition, through generation of excess nitric oxide (NO), palmitate causes mitochondrial DNA damage-induced apoptosis in INS-1 cells [91]. Palmitate was also reported to trigger ROS production from diverse sources including the mitochondrial electron transport chain [92], peroxisomes [93], or due to NADPH oxidase activation [94]. A recent transcriptomic/proteomic profiling using INS-1E cells and isolated human islet data further suggested that palmitate may elicit an oxidative stress response in beta-cells [86].

#### 2.2.3. Autophagy and Ubiquitin–Proteasome System Impairment

Palmitate was firstly suggested to stimulate autophagy in INS-1 cells [95], but other studies revealed that exposure to palmitate rather impairs lysosomal degradation in pancreatic beta-cells [78,96,97,98]. In human islets chronically exposed to palmitate, beta-cells present a massive increase in autophagic vacuoles and autophagosomes associated with decreased lysosomal-associated membrane protein 2 (LAMP2) and cell death, similar to T2D islets [55]. These observations suggest that palmitate alters autophagic removal of these structures. Indeed, elevated levels of palmitate were shown to increase autophagosome numbers [78,96,97,99,100,101] but alter autophagic flux in clonal beta-cells and human islets [78,96,97,98]. Among the mechanisms involved in palmitate-induced autophagic flux impairment, defect in lysosomal acidification and function [96,98], activation of mammalian target of rapamycin complex 1 (mTORC1), an inhibitor of autophagy [97], and ER stress-induced JNK activation [99] were proposed to contribute to this lipotoxic alteration. In addition, RNA-sequencing analysis of palmitate-treated human islets reveals a decrease in autophagy-related and lysosomal function-related genes that may affect autophagosome–lysosome fusion [78]. The link between palmitate-induced autophagy alteration and beta-cell apoptosis was evidenced by the use of autophagy enhancing drugs such as rapamycin and carbamazepine. Despite some controversies regarding the role of palmitate on autophagy modulation (inhibition or activation), all studies unanimously reported that stimulation of autophagy restores autophagic flux and decreases palmitate-induced apoptosis in rodent beta-cells and human islets [78,96,97,101,102,103], while blocking autophagy exacerbates beta-cell lipoapoptosis [102]. Supporting the relevance of these findings in human T2D, rapamycin was shown to restore autophagic flux and to alleviate ER stress and beta-cell death in human T2D islets [102], further pointing to a protective role of autophagy in the maintenance of beta-cell integrity.

The role of FFAs on the ubiquitin–proteasome system has been less investigated. However, studies reported that palmitate disrupts the proteasome function as demonstrated by the altered expression of genes associated with proteasome activity in human islets [78] and the accumulation of ubiquitinated proteins in the MIN6 beta-cell line, isolated mouse and human islets, similar to what is observed in pancreatic sections from mice fed a high fat diet and from obese human donors [88]. Under these conditions, activation of the proteasome decreases ubiquitinated proteins and prevents the proapoptotic pathway induced by palmitate in MIN6 cells [88].

#### 2.2.4. Inflammation

Supporting a role of lipotoxicity in islet inflammation, several studies revealed that exposure of human islets to palmitate promotes the expression of proinflammatory cytokines and chemokines [104,105]. Whereas this IL1β-dependent induction of cytokines and chemokines can be prevented by IL1R antagonism [104,105], blockage of IL1β signaling does not protect human islets from lipotoxicity-induced beta-cell death [105], suggesting that palmitate-induced mild inflammation may not be involved in beta-cell apoptosis. In addition, Wali et al. demonstrated that palmitate-induced islet cell death is not dependent on the activation of the NLRP3 inflammasome [106]. Recent studies however revisited the role of palmitate-induced inflammation in beta-cell apoptosis. Indeed, Hu et al. identified the stimulator of interferon genes-interferon regulatory factor 3 (STING-IRF3) as a novel signaling pathway involved in lipotoxicity-induced beta-cell inflammation and apoptosis using INS-1 cells and islets from *db*/*db* mice [107]. Furthermore, palmitate exposure also triggers secretion of a member of damage-associated molecular patterns (DAMPs) by isolated human islets to promote macrophage infiltration of the islets, further driving islet inflammation and beta-cell apoptosis [108]. Significant increase in the expression of this specific DAMPs molecule was detected in islets of *db*/*db* mice, highlighting the potential relevance of this mechanism in vivo [108].

### 2.3. Glucotoxicity and Glucolipotoxicity

Once the pathogenesis of diabetes is established, the sustained elevated levels of glucose seen in individuals with T2D may ultimately exacerbate the loss of functional beta-cells, and this concept has been termed “glucotoxicity” [109]. Supporting this assumption, high glucose exposure has been shown to trigger beta-cell apoptosis in cultured human islets [110,111,112]. In the course of obesity-associated T2D, the combined excess of glucose and lipids may synergize to cause a faster and severe progression of beta-cell deficit, a phenomenon called “glucolipotoxicity” [113,114,115,116], albeit debated [74]. In this section, we will report recent mechanisms involved in glucotoxicity-induced beta-cell apoptosis as well as the deleterious effects of glucolipotoxicity.

#### 2.3.1. ER Stress and Aberrant Ca^2+^ Release

Chronic hyperglycemia was shown to perturb ER homeostasis and to induce ER stress in pancreatic beta-cells. Indeed, prolonged exposure to high glucose leads to Ca^2+^ efflux from the ER to the cytosol, a process ultimately involved in beta-cell death [82]. This deleterious Ca^2+^ efflux from the ER was explained by the downregulation of the sarco/endoplasmic reticulum Ca^2+^-ATPase 2b (SERCA2b) pump as observed in INS-1 832/13 cells treated with chronic high glucose, but also in islets from *db*/*db* mice and human islets from subjects with T2D [82]. Importantly, the ER Ca^2+^ depletion is worsened in INS-1 832/13 beta-cells exposed to glucolipotoxic conditions [82]. Recently, a phenotypic screen conducted to identify molecules that protect beta-cells further points to Ca^2+^ overload as a key mechanism of glucolipotoxicity-induced apoptosis in INS-1E cells, rat and human islets [117]. In addition, gluco(lipo)toxicity has been shown to induce ER stress-mediated beta-cell apoptosis through the induction of the PERK-dependent proapoptotic factor CHOP in mouse islets [118]. mTORC1 also appears as an important transducer of ER stress response under glucolipotoxicity as demonstrated by its implication in the activation of IRE1α-JNK pathway [119]. Glucotoxicity-mediated ER stress further induces activation of apoptosis-initiating Bcl-2 homology domain 3 (BH3) proteins such as Bim and PUMA in mouse islets [118], pointing to the reciprocal interplay between ER stress and mitochondrial pathway of apoptosis under nutrient stress conditions.

#### 2.3.2. Oxidative Stress and Mitochondrial Dysfunction

Exposure to high levels of glucose (in combination with FFAs) has been shown to affect beta-cell viability by inducing oxidative stress and mitochondrial apoptosis [116,120]. Indeed, inhibition of oxidative stress protects clonal beta-cells, mouse islets and human islets against the adverse effects of glucotoxicity [118,120]. Recently, altered iron metabolism has been identified as a novel mechanism relaying gluco(lipo)toxicity to cytosolic ROS production, mitochondrial dysfunction and beta-cell apoptosis in isolated mouse islets, but also in vivo in a transgenic mouse model with beta-specific knockout of an iron transporter [121]. Supporting the involvement of mitochondrial dysfunction in glucotoxicity-induced beta-cell demise, a global downregulation of mtDNA-encoded respiratory chain subunits has been shown in human islets chronically exposed to elevated glucose levels [110]. This alteration may lead to altered respiratory activity and increased susceptibility of beta-cells to apoptosis [110]. Furthermore, elevated glucose (and FFAs) alters mitochondrial dynamics in INS-1 cells [90] and in islets from diabetic Goto Kakizaki (GK) rats [89,122], whereas its preservation protects INS-1 beta-cells from glucolipotoxicity-induced mitochondrial fragmentation and apoptosis [90].

#### 2.3.3. Autophagy and Ubiquitin–Proteasome System Impairment

Chronic exposure to elevated glucose has been shown to favor accumulation of ubiquitinated proteins in clonal rat beta-cells and human islets [123,124]. Large aggregates of ubiquitinated proteins in beta-cells were also observed on Zucker diabetic fatty rat pancreatic sections [124]. These observations suggest that the degradation systems removing such modified proteins (autophagy and/or proteasome) are dysfunctional in beta-cells exposed to glucotoxic conditions. In Broca et al., accumulation of ubiquitinated proteins was attributed to a decrease in proteasomal function as shown in high glucose-treated INS-1E cells, human islets and hyperglycemic GK rat islets [123]. The alteration in proteasomal activity was shown to be involved in ER stress induction and subsequent beta-cell apoptosis in INS-1E cells and human islets [123]. In contrast, it was also reported that although the proteasome is recruited to ubiquitinated protein aggregates in clonal beta-cells exposed to high glucose, autophagy is rather involved in mediating their clearance [124], therefore suggesting an alteration of beta-cell autophagic clearance under glucotoxic treatment. Only few studies investigated the role of glucotoxicity on beta-cell autophagy. Glucotoxicity was shown to positively regulate autophagy via PTEN-induced putative kinase 1 (PINK1) in INS-1 and rat beta-cells [125] with an increased number of autophagosomes detected in human islets [110], but it was also suggested that glucotoxicity alters lysosomal degradation in human islets [97]. Most studies investigated the effects of glucolipotoxicity and revealed that whereas glucose and palmitate synergize to increase autophagosome formation [56,96,97,126], this combination also impairs autophagic flux through lysosomal dysfunction in clonal beta-cells, mouse and human islets [56,96,97]. This defect occurring downstream of ER stress leads to accumulation of defective lysosomes and subsequent release of hydrolytic enzymes such as cathepsin D through lysosomal membrane permeability [56]. This release of cathepsin D into the cytoplasm, observed in treated INS-1E cells, mouse islets and pancreatic sections from T2D subjects, is involved in glucolipotoxicity-induced beta-cell death [56]. To further support the involvement of defective autophagic clearance as a mediator of glucolipotoxicity, stimulation of autophagy with rapamycin was shown to protect rodent clonal beta-cells from glucose and lipid excess-induced apoptosis [97,126].

#### 2.3.4. Inflammation

Whereas intra-islet IL1β production was suggested to be a toxic response to high glucose exposure in human islets [127,128], other studies failed to demonstrate any increase in IL1β expression in human beta-cells exposed to glucotoxicity [105,106,129]. Furthermore, neither IL1β production nor activation of the NLRP3 inflammasome complex seem to mediate islet cell death in response to glucotoxicity or glucolipotoxicity [106,129,130,131]. Linking glucotoxicity to islet inflammation, a recent study, however, reported that high glucose concentrations trigger secretion of a signaling molecule (DAMPs) from pancreatic human islets, promoting macrophage infiltration of the islets, further driving islet inflammation and beta-cell apoptosis [108]. This cascade of events is exacerbated under a combination of glucose and lipids [108]. Attraction of circulating immune cells involved in local islet inflammation and beta-cell death may also be mediated by gluco(lipo)toxic activation of the transcription factors nuclear factor-kappa B (NFκB) and signal transducer and activator of transcription 1 (STAT1) through tumor necrosis factor receptor 5 (TNFR5) induction, as shown in INS-1 cells, human islets, and islets from high fat-fed mice [132].

#### 2.3.5. Epigenetic Mechanisms and Nuclear Events

Epigenetic mechanisms may also contribute to gluco(lipo)toxicity-induced beta-cell apoptosis by modulating gene expression through chromatin modification and/or non-coding RNAs. Indeed, Li et al. demonstrated that the microRNAs (miRNAs) miR-375, miR-30a and miR-34a are increased in INS-1 cells and pancreatic islets exposed to high glucose levels, as well as in islets from diabetic GK rats [133]. miRNAs are endogenous non-coding RNAs known to regulate gene expression by binding to the 3′UTR of their target mRNAs resulting in their degradation and/or translational inhibition. In this study, glucotoxicity-induced miR-375, miR-30a and miR-34a are involved in the inactivation of Notch1 pathway, resulting in INS-1 cell apoptosis [133]. Using a miRNA microarray analysis, another study identified a set of differentially expressed miRNAs in human islets exposed to glucolipotoxic conditions [134]. Among them, miR-299-5p was shown to be downregulated and this was revealed as a key mediator of glucolipotoxicity-induced beta-cell apoptosis in human islets [134]. Regarding the factors involved in chromatin remodeling, the histone acetyl-transferase p300 was shown to be diminished in clonal beta-cells and human islets exposed to gluco/lipotoxicity as well as in beta-cells of human T2D donors [135]. This study further demonstrates that alteration of p300 levels and activity plays a key role in mediating apoptosis in INS-1E cells and isolated mouse islets [135]. The transcription factor cAMP-responsive element-binding protein (CREB) is another key component of the transcriptional beta-cell machinery promoting cell survival. We demonstrated that the degradation of CREB by the ubiquitin-proteasome system is a mechanism subserving glucotoxicity-induced beta-cell death in rodent beta-cells and human islets [136]. Finally, induction of the abovementioned deleterious mechanisms in gluco(lipo)toxic INS-1 832/13 cells, rodent and human islets culminates in activation of executioner caspases (-3 and -6) that are involved in the degradation of nuclear lamins, components of the lamina in the nuclear envelope [137,138]. As a consequence of lamin degradation, chromatin condensation and collapse of nuclear envelope under glucotoxicity appear as additional events involved in beta-cell demise [137,138].

## 3. Molecular Mechanisms Induced by GLP-1 to Protect Beta-Cells from Apoptosis

Oral glucose induces a greater stimulation of insulin secretion than intravenous glucose administration. This is called the incretin effect [139] and is caused by the release of GLP-1 and glucose-dependent insulinotropic polypeptide (GIP) from the gut by L and K cells, respectively. GIP has a poor insulinotropic efficacy in T2D, thus incretin based-therapies have been focused on GLP-1. Moreover, GLP-1 based therapies have positive impact in promoting weight loss [140] and present low risk of hypoglycemia as they induce insulin secretion in the presence of elevated glucose concentrations. GLP-1 has a short half-life (1–2 min) as it is rapidly degraded in the circulation by a serine exopeptidase dipeptidyl peptidase 4 (DPP-4) [139]. Consequently GLP-1 receptor agonist (GLP-1RA) therapies have been developed in generating either stable derivatives of GLP-1 with prolonged action resistant to DPP-4 (liraglutide, dulaglutide, semaglutide, lixisenatide) [140], or derivatives of exendin-4 (exenatide, lixisenatide) [140]. Instead, another strategy consists of using inhibitors of DPP-4 (iDPP-4) activity to preserve the endogenous production of GLP-1 [141].

At the molecular level, GLP-1 binds to its receptor (GLP-1R) belonging to the G-protein coupled receptor (GPCR) family, which is known to be positively coupled to cAMP production, albeit a coupling to Gq has also been reported [142,143]. The intracellular increase in cAMP production activates protein kinase A (PKA) and cAMP-activated guanine nucleotide exchange factors that target Ras-like GTPases 2 (EPAC2), which in turn mediate changes in ion-channel activity leading to an increase in cytosolic Ca^2+^ concentration, but also exert a direct action at the level of the exocytosis machinery [144] (Figure 2). These events enhance the stimulation of insulin secretion in a glucose-dependent manner. The GLP-1R is also known to recruit scaffold proteins such as beta-arrestins (ARRBs) [145,146] that may activate extracellular signal-related kinases 1 and 2 (ERK1/2) [147,148] or c-SRC [149], which are known to be involved in beta-cell survival. Of note, ERK1/2 are also activated by GLP-1 independently of ARRBs in a PKA dependent manner [147].

### 3.1. GLP-1RA Alleviates Beta-Cell Apoptosis Induced by Diabetogenic Conditions or in T2D

GLP-1R activation triggers the transcription of genes involved in proliferation, or that display antiapoptotic and/or anti-inflammatory activities in beta-cells, suggesting that GLP-1 promotes beta-cell survival and regulates beta-cell mass [150]. However, whereas a treatment with long lasting analogues such as liraglutide or dulaglutide are able to promote pancreatic beta-cell proliferation in diabetic *db*/*db* mice [151,152], in high-fat-fed and streptozotocin-induced [153] or alloxan-induced [154] mouse model of T2D, it is well accepted that adult human beta-cells have a limited capacity to proliferate [155]. On the contrary, many studies have identified in rodent and human that GLP-1RA alleviate beta-cell apoptosis [151,156,157,158,159] induced by several stressors such as gluco/lipotoxicity [160,161,162,163], which may trigger oxidative [164] and ER stress [162,165], or by cytokines [166,167,168,169]. A reduced beta-cell apoptosis was also observed in diabetic *db*/*db* [152] and Akita [170,171] mice or prediabetic GK rats [172] chronically treated with GLP1-RA. Unlike GLP-1-induced insulin secretion, its role as a pro survival molecule is far from a consensus, and pleiotropic effects have been reported. Indeed, multiple signaling pathways and the regulation of various genes have been described. Therefore, our knowledge regarding the molecular mechanisms involved is unclear. For instance, depending on studies and models, GLP-1RA were shown to protect beta-cells from apoptosis through PKA and phosphoinositide 3-kinase (PI3K)-AKT-dependent pathways partly via the inhibition of ER stress [162,165,171,173] by increasing immunoglobulin heavy-chain binding protein (BiP) and JunB [162] or by blocking the induction of sterol regulatory element-binding protein 1 (SREBP1c) and C/EBPb transcription factors [174]. Independently of ER stress, PKA and PI3K-AKT-dependent pathways were shown to be involved in suppression of Forkhead box O1 (FoxO1) [160,175,176], the restoration of pancreatic and duodenal homeobox 1 (PDX1) [176,177] via mammalian sterile 20-like kinase 1 (Mst1) inactivation [161], inactivation of NADPH oxidase 2 (NOX2) [163] or improved mitochondrial function [161] by suppressing sustained AMP-activated protein kinase (AMPK) hyperactivation [178]. Exendin-4 also reduced oxidative damage and apoptosis through Ca^2+^-independent phospholipase A2 [179], or preserved proteasome activity from the deleterious effects of glucotoxicity [123] in clonal beta-cell lines.

Other signaling that is known to be involved in beta-cell survival is also improved upon GLP-1R treatment such as the insulin signaling [180] or the activation of the transcription factor CREB [181,182] leading to the expression of the insulin receptor substrate 2 (IRS-2) [181,182]. Finally, GLP-1 was shown to trigger the phosphorylation of Bad at specific Ser sites leading to its inactivation, via SAD-A, a serine/threonine protein kinase of the AMPK subfamily [183] or via ERK1/2- phosphorylated p90 ribosomal S6 kinase (p90RSK) activation [147]. On the contrary, a prolonged treatment with GLP-1 did not retain protective effect probably because of the increased ER stress [160].

Autophagy, which prevents beta-cell injury and death by protecting against ER stress, inflammation and/or oxidative stress, was shown to be affected by GLP1-RA. Exendin-4 was primarily reported to improve beta-cell function and survival without modulating the autophagic flux [184]. This was demonstrated by only measuring p62 expression in diabetic *db*/*db* mice [184]. Nevertheless, with the development of new techniques to monitor the autophagic flux, it is now agreed that GLP-1RA may indeed modulate autophagy in beta-cells [184]. In glucolipotoxic beta-cells (clonal INS-1E cells and human islets) that showed increased apoptosis, the number of autophagosomes was shown to be increased, demonstrating a blockade of the autophagic flux [56]. Interestingly, treatment of beta-cells and/or animals with exendin-4 [56] or liraglutide [153,185,186,187] rescued lysosomal function and autophagic flux in both lipotoxic and glucolipotoxic conditions leading to a protective effect on beta-cells, and suggesting that stimulation of the autophagic flux by GLP-1 is critical for its protective effects [188].

The mechanisms underlying the impact of GLP-1RA on both autophagy and lysosomal function are not yet elucidated, but several pathways have been explored. It has been recently reported in INS-1 cells that liraglutide ameliorated the injury triggered by lipotoxic conditions through the upregulation of autophagy mediated by FoxO1 [187], or throughout the upregulation of mesencephalic astrocyte derived neurotrophic factor (MANF) in MIN6 cells [189], thus protecting cells from ER stress. It has also been reported that GLP-1 may protect beta-cells from glucotoxicity through enhancing autophagy by AMPK inhibition in INS-1 cells [190]. GLP-1-induced protection against apoptosis through the autophagic flux in human beta-cells still need to be fully explored.

Only a limited number of studies have investigated the potential impact of GLP-1RA on IAPP toxicity. Exendin-4 alleviated h-IAPP-induced apoptosis in MIN6 [191] and in INS-1E [192] beta-cell lines, in islets from h-IAPP transgenic mice [193] or in human islets [194,195]. The protection was not associated with a reduced formation of h-IAPP deposits [192], but with increased levels of AKT phosphorylation [192,193,194,195]. Several mechanisms downstream AKT phosphorylation have been reported such as reduced IL1β immunoreactivity and release [194], the enhancement of pro h-IAPP processing [195], translocation of PDX-1 in the nucleus [192] and improved mitochondrial function [192]. A reduced JNK activation has not always been noticed [192,195], and exendin-4 protection from h-IAPP toxicity does not seem to alleviate ER stress in INS-1E cells [192]. Finally, an improvement of the autophagic flux by exendin-4 was reported in MIN6 cells overexpressing h-IAPP [191].

### 3.2. iDPP-4 Alleviates Beta-Cell Apoptosis Induced by Diabetogenic Conditions

Although the impact on weight loss is less pronounced than that of GLP-1RA, inhibition of DPP-4 activity is another potent strategy for preserving both GLP-1 and GIP endogenous production, and therefore enhancing incretin-induced insulin secretion in T2D [141]. Few studies in vivo have investigated the impact of DPP-4 inhibition on beta-cell survival [196,197,198,199,200,201,202]. In Zucker diabetic rats, the plasma levels of GLP-1 were increased upon alogliptin treatment, and beta-cell survival was improved through CREB activation, and restoration of Bcl-2 and IRS-2 expression [196]. Vildagliptin reduced beta-cell apoptosis in a mouse model of diabetes (KK-Ay-TaJcl) [197] and in *db*/*db* [199] mice, and this was associated with decreased ER [197,199] and oxidative stress [197]. Moreover, vildagliptin was also reported to protect beta-cells from inflammation in advanced-aged diet-induced obesity mouse model [200], while a treatment with another iDPP-4, MK-626, improved the autophagic flux in high fat diet-induced obese mice [201].

In addition to preserving GLP-1 secreted by the gut, recent studies have reported that inhibiting DPP-4 may also protect GLP-1 released locally by islets [203,204,205,206]. Indeed, DPP-4 is expressed in rodent and human islets [205,207,208,209,210] and GLP-1 was reported to be expressed [205,211,212] and released [205,211,212] by islet alpha-cells upon glucose [211], arginine [211], GPR142 activation [213] or GIP stimulation [214]. Intra-islet GLP-1 makes a significant contribution to islet adaptation, particularly expansion of beta-cell mass to face insulin resistance [158,215] or adaptation in pregnancy [216] in mice. Moreover, intra islet GLP-1 was shown to reduce apoptosis triggered by lipotoxicity [158] and glucolipotoxicity [213] in rodent beta-cells, whereas blocking GLP-1R signaling in beta-cells with exendin-(9–39) decreased cell viability and increased cell apoptosis via PDX1 inhibition [158]. Inhibiting DPP-4 activity in human beta-cells protected against gluco- [203], lipo- [203] and cytokine- [203,204] induced toxicity by reducing cytokine production and secretion from islets [203] and NFκB1 expression [204]. A reduction of oxidative stress was also involved [203]. Most importantly, it also reduced apoptosis in islets from T2D donors, suggesting that inhibiting DPP-4, besides playing a role in incretin effects, directly affects beta-cell survival [204]. Nevertheless, it should be stressed that in T2D islets the proportion of alpha-cells expressing GLP-1 is increased [205] while DPP-4 expression [204,210] and activity [208] are reduced, leading to an increased secretion of GLP-1 [211]. Therefore, a protective role of iDPP4 in preserving intra islet GLP-1 needs to be further addressed to unequivocally determine its relevance in T2D.

## 4. Conclusions and Perspectives

This review relates the molecular mechanisms involved in beta-cell apoptosis. As stated above for clarity, we independently described the stress pathways involved, but it has to be considered that crosstalk between these pathways may occur at different levels to further exacerbate beta-cell death (Figure 1). Moreover, even though amyloid deposits, lipotoxicity and glucotoxicity are the “most investigated” causative factors of beta-cell demise, hostile environmental context may also superimpose deleterious mechanisms to further accelerate the progression towards overt T2D. Indeed, a new area of research points to the detrimental consequences of altered circadian rhythms/sleep deprivation or exposure to pollutants on beta-cell survival [217,218,219].

It should also be stressed that mainly in vitro or ex vivo molecular mechanisms involved in beta-cell apoptosis were reported as stated in the introduction. Although studies in human islets have been described, the relevance of these mechanisms remains to be proven in T2D. In particular, whether “lipotoxicity” has a significant deleterious effect on beta-cells in vivo. Another key point that is still unknown, is whether GLP-1RA-based therapies in T2D patients participate in the protection and maintenance of the beta-cell mass in vivo. This question is unresolved because we are still lacking technologies to assess in 3D the real beta-cell mass in vivo in humans.

Finally, whereas the protective mechanisms of GLP-1R based therapies have been studied, only few studies have investigated beta-cell protection induced by the other incretin GIP [220], and its relevance in human islets remains to be established. This is a critical question that will undoubtedly be addressed in futures studies. Indeed, although GLP-1 and GIP have some overlapping functionality, their combined use (dual agonist also called twincretins) leads to synergistic effects on diabetes and related metabolic disease [221,222,223]. Therefore, development of dual agonists and elucidation of their potential role on beta-cell mass preservation represent a considerable interest to improve current GLP-1R-based therapies in T2D.
